# Effect of p38 inhibitor on the proliferation of chicken muscle stem cells and differentiation into muscle and fat

**DOI:** 10.5713/ab.22.0171

**Published:** 2022-09-02

**Authors:** Minkyung Ryu, Minsu Kim, Hyun Young Jung, Cho Hyun Kim, Cheorun Jo

**Affiliations:** 1 Department of Agricultural Biotechnology, Center for Food and Bioconvergence, and Research Institute of Agriculture and Life Science, Seoul National University, Seoul 08826, Korea; 2 Institute of Green Bio Science and Technology, Seoul National University, Pyeongchang 25354, Korea

**Keywords:** Adipogenesis, Chicken, Myoblast, Myogenesis, Satellite Cell

## Abstract

**Objective:**

Inhibiting the p38 mitogen-activated protein kinase (MAPK) signaling pathway delays differentiation and increases proliferation of muscle stem cells in most species. Here, we aimed to investigate the effect of p38 inhibitor (p38i) treatment on the proliferation and differentiation of chicken muscle stem cells.

**Methods:**

Chicken muscle stem cells were collected from the muscle tissues of Hy-line Brown chicken embryos at embryonic day 18, then isolated by the preplating method. Cells were cultured for 4 days in growth medium supplemented with dimethyl sulfoxide or 1, 10, 20 μM of p38i, then subcultured for up to 4 passages. Differentiation was induced for 3 days with differentiation medium. Each treatment was replicated 3 times.

**Results:**

The proliferation and mRNA expression of paired box 7 gene and myogenic factor 5 gene, as well as the mRNA expression of myogenic differentiation marker gene myogenin were significantly higher in p38i-treated cultures than in control (p<0.05), but immunofluorescence staining and mRNA expression of myosin heavy chain (*MHC*) were not significantly different between the two groups. Oil red O staining of accumulated lipid droplets in differentiated cell cultures revealed a higher lipid density in p38i-treated cultures than in control; however, the expression of the adipogenic marker gene peroxisome proliferator activated receptor gamma was not significantly different between the two groups.

**Conclusion:**

p38 inhibition in chicken muscle stem cells improves cell proliferation, but the effects on myogenic differentiation and lipid accumulation require additional analysis. Further studies are needed on the chicken p38-MAPK pathway to understand the muscle and fat development mechanism.

## INTRODUCTION

Cultured meat has emerged as a popular meat alternative to meet the increasing demand for muscle food sources [1,2]. Cultivated meat production begins with the selection of a cell source, followed by the extraction of myogenic cells from these sources, which are then cultured and grown on a larger scale before being differentiated into muscle tissue [3]. Thus, there is a pressing need to upscale the proliferation of myogenic cells as much as possible, while not losing myogenic differentiation abilities for the production of cultured meat on a commercial scale.

The sourcing of cells may be achieved in two ways: by isolating primary cells from live stock muscle tissues such as satellite cells, also known as muscle stem cells, or using pluripotent cell sources such as embryonic stem cells or induced pluripotent stem cells (iPSCs) [4]. While iPSCs may potentially proliferate indefinitely and can differentiate into a wide range of cell types, it is costlier and more difficult to culture or induce their differentiation into the desired cell type than it is for satellite cells. In contrast, satellite cells have a limited expansion capability but are more accessible and can more easily differentiate into myotubes and myofibrils than iPSCs, thus, making them a popular choice over iPSCs in the cultured meat industry [5].

Satellite cells are adult stem cells found in the skeletal mus cle. They can contribute to muscle regeneration by symmetric and asymmetric division [6]. Symmetric division gives rise to 2 committed myoblast progenitors which may fuse into damaged muscle fibers. Asymmetric division results in the formation of two different daughter cells, one of which remains quiescent and the other of which commits to the myogenic fate. The latter, under specific circumstances, differentiates and matures into myotubes, which then repair muscle tissue. During this process, various transcription factors that are commonly used as markers of myogenesis, proliferation, and differentiation in satellite cells are expressed [7–9]. At the quiescent stage, satellite cells express paired box 7 (Pax7) but not myoblast determination protein 1 (MyoD) or myogenic factor 5 (Myf5). Upon activation through asymmetric division, committed satellite cells begin to express MyoD, which regulates cell proliferation and differentiation. As cells start to undergo differentiation, Pax7 expression is depleted. Differentiation of these myoblasts into myotubes is indicated by the expression of myogenin (MyoG) and myosin heavy chain (MHC).

As mentioned above, satellite cells can undergo myogenic differentiation more easily than iPSCs; however, the limitation in cell proliferation over cell aging is a setback for mass production of cultured meat, as well as *in vitro* studies on muscle regeneration. Thus, previous studies on both cultured meat production and muscle regeneration have focused on avoiding cell senescence and maintaining stemness, which is indicated by the prolonged expression of Pax7 [10–12]. The p38-mitogen-activated protein kinase (MAPK) signaling pathway is a major regulatory pathway that induces myogenic differentiation upon muscle injury [13]. Previous studies across various species, such as humans, cattle, and pigs, have shown that activation of the p38-MAPK pathway induces cell senescence, while inhibition of p38 maintains cell proliferation [10,11,14]. However, the majority of studies focus on myoblasts of mammalian origin. While the general myogenic process may be similar, we believe there is still a need to differentiate between the cellular responses of mammalian and avian myoblasts [15]. For example, one study states that dystrophic muscle fibers in avian species display more variability in their physiological features compared to normal muscle fibers, and another study reports that satellite cell lineages regarding contractile isoforms are fixed in chicks, while embryonic mammalian satellite cells may transform into different lineages [16,17]. Adipose tissue may also hold different characteristics between species, as uncoupling protein 1, which is present in brown adipose tissue of mammals, is not found in avian species [18]. Nevertheless, studies on the effect of the p38 inhibitor have been conducted mostly on mammalian myoblasts and not on avian myoblasts. Thus, we aimed to maintain the stemness of chicken muscle stem cells by inhibiting the p38-MAPK pathway.

In this study, we also found that treatment with the p38 pathway inhibitor induced the formation of lipid droplets in muscle stem cell cultures. This may be due to the transdifferentiation of myoblasts to adipocytes, as reported in previous studies [19,20].

## MATERIALS AND METHODS

### Animal care

Fertile Hy-line Brown chicken eggs were purchased from a local farm. The maintenance and experimental procedures for eggs and embryos were approved by the Institutional Animal Care and Use Committee (IACUC) at Seoul National University (Approval no.: SNU-210310-6). The experiments were performed according to the standard protocol of the Institute of Laboratory Animal Resources at Seoul National University.

### Isolation, culture, and differentiation of chicken muscle stem cells

Fertile chicken eggs were incubated at 38°C until embryonic day 18 (E18) and then euthanized with CO_2_. Muscle tissues from the breasts of 16 chicken embryos were collected and washed with Dulbecco’s phosphate-buffered saline (DPBS; Welgene, Gyeongsan, Korea) containing 2× antibiotic–antimycotic (AA; Gibco, Gaithersburg, MD, USA). Fat, connective tissues, and blood vessels were removed from the muscle tissues. Collected muscle tissues were minced with scissors to form a slurry and then digested in twice the volume of 0.8 mg/mL pronase (Sigma-Aldrich, St. Louis, MO, USA) in DPBS for 40 min at 37°C with vortexing every 10 min. The digested slurry was then filtered through a 100 μm and 40 μm cell strainer sequentially and centrifuged at 1,200×*g* for 3 min. The supernatant was removed, and the resulting cell pellet was collected.

#### Isolation of chicken muscle stem cells

Muscle stem cells were isolated by the pre-plating method and the collected cell pellet was reconstituted in Minimum Essential Medium, Alpha modification with 10% fetal bovine serum (FBS; Gibco, USA) and pre-plated on surface-treated cell culture plates for 1 h. The supernatant, containing muscle stem cells, was collected and centrifuged at 1,200×g for 3 min. The cell pellet was reconstituted in growth media (GM; Ham’s F-10 [Welgene, Korea] containing 10% [v/v] FBS, 1×Glutamax, 1×AA, and 0.1 mM β-mercaptoethanol [all from Gibco, USA]) and cultured for 3 days until 90% confluency on cell culture plates coated with 0.1% gelatin. Cells were then dissociated from the plates using 0.04% trypsin, counted, and cryopreserved in GM containing 10% dimethyl sulfoxide (DMSO) until use.

#### Culture and differentiation of muscle stem cells

Chicken muscle stem cells were cultured in GM supplemented with either DMSO or 1, 10, or 20 μM SB203580 (Cayman Chemical, Ann Arbor, MI, USA) at a 1:1,000 ratio. Cells were seeded at 5.0×10^4^ cells per well and then dissociated by treatment with 0.04% trypsin on day 4 of culture. Dissociated cells were counted with a hematocytometer to determine cell density before being subcultured at the same seeding density for up to four passages.

For the analysis of differentiated cells, cells were first cul tured in GM for 4 days at each passage, and the culture medium was replaced with differentiation medium (DM; Dulbecco’s modified eagle’s medium [Welgene, Korea] containing 1×Glutamax, 1×AA, 0.1 mM β-mercaptoethanol, and 1% KnockOut serum replacement [Gibco, USA]). Then, the cells were allowed to differentiate for 3 days. All media were changed every other day, and the cells were cultured in a humidified cell incubator at 5% CO_2_ at 37°C.

### Immunofluorescence staining

Muscle stem cells on day 4 of culture or day 3 of differentiation were fixed in 4% paraformaldehyde in DPBS for 30 min at 4°C. Fixed cells were washed twice with DPBS and then permeabilized with 0.2% (v/v) Triton X-100 (Sigma-Aldrich, USA) for 15 min before blocking non-specific binding with 10% (v/v) goat serum (Thermo Fisher Scientific, Waltham, MA, USA) for 1 h. Primary antibodies against chicken Pax7 (1:200; R&D Systems, Minneapolis, MN, USA) or myosin heavy chain (1:1,000; R&D Systems, USA), respectively, were added, and the cells were incubated at 4°C overnight. Goat serum and primary antibodies were removed, and cells were incubated with secondary antibodies (1:1,000; Invitrogen, Waltham, MA, USA, A -11001) at 4°C overnight. The nuclei were stained with Hoechst 33342 (1:1,000; Molecular Probes, Eugene, OR, USA) for 10 min at 4°C, and the cells were washed, followed by media replacement with DPBS. Stained images were captured using an inverted fluorescence microscope.

### Oil red O staining

Muscle stem cells on day 3 of differentiation were fixed in 4% paraformaldehyde in DPBS for 30 min at 4°C. The oil red O working solution was prepared by mixing the oil red O stock solution with double distilled water at a 3:2 ratio. The fixed cells were washed twice with water and covered with 1 mL of the oil red O working solution. After 15 min of incubation, the working solution was removed, and the cells were washed five times with water. Stained cells were covered with water, and the images were captured using an inverted fluorescence microscope.

### Quantitative reverse-transcription polymerase chain reaction (qPCR)

Total RNA was extracted from muscle stem cells on day 4 of culture or day 3 of differentiation using TRIzol reagent (Invitrogen, Carlsbad, CA, USA), and cDNA was synthesized using the high-capacity RNA-to-cDNA Kit (Applied Biosystems, Foster City, CA, USA), following the manufacturer’s instructions. cDNA was amplified using a DyNAmo HS SYBR Green qPCR Kit (Thermo Fisher Scientific, USA) containing 1 to 2 pmol of each primer set in a 10 μL reaction volume. The primer sequences are listed in Table 1. Amplification and detection were performed using the ABI 7300 Real-Time PCR System (Applied Biosystems, USA) under the following conditions: one cycle at 50°C for 2 min and 95°C for 10 min, followed by 40 cycles of denaturation at 95°C for 15 s and annealing/extension at 60°C for 1 min. The dissociation curves were analyzed, and the amplified products were loaded onto gels to confirm the specificity of the polymerase chain reaction (PCR) products. Relative expression levels were calculated by normalizing the threshold cycle (Ct) values of each gene to the reference gene, glyceraldehyde-3-phosphate dehydrogenase, using the delta-delta Ct method.

### Statistical analysis

A general linear model analysis was performed using SAS software (version 9.4, SAS Institute Inc., Cary, NC, USA). Significant differences between groups were determined by Tukey’s multiple range test with a confidence level of p<0.05.

## RESULTS

### Treatment of p38i increases the proliferation of chicken muscle stem cells

**Figure 1:**
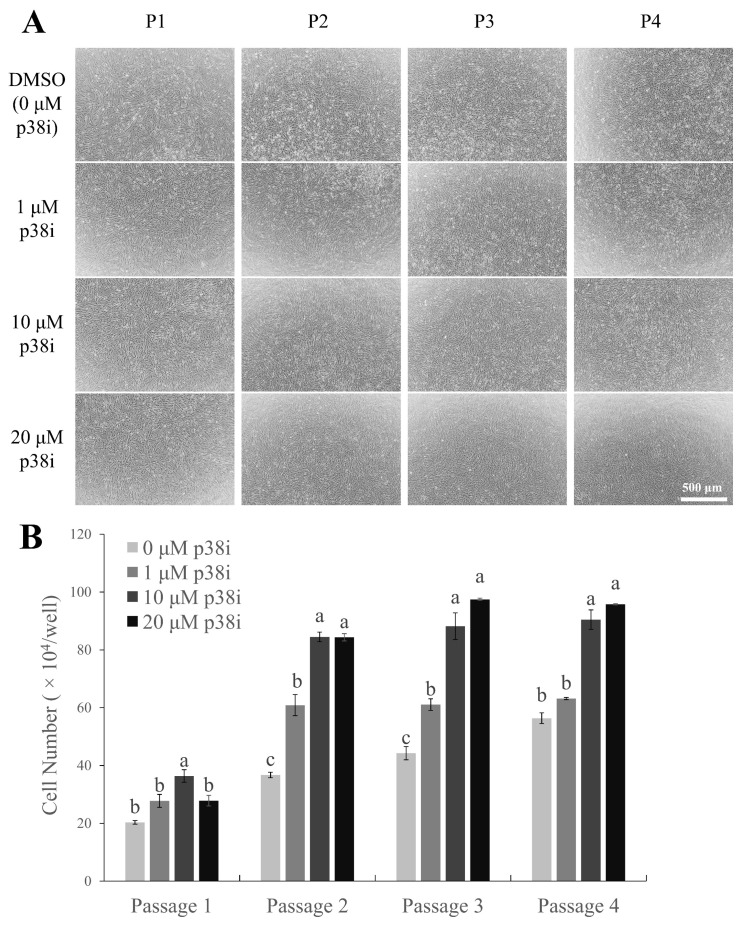
Effect of p38i on the proliferation of chicken muscle stem cells. (A) Morphology of cultured cells. (B) Cell number of control and p38i-treated muscle stem cell cultures. Cells were counted on day 4 of proliferation. All passages of each replication originated from the same primary cell line. p38i, p38 inhibitor. ^a–c^ Different letters within the same passage number indicate significant differences (p<0.05). Scale bar = 500 μm.

Chicken muscle stem cells were cultured with p38i- or DMSO-treated growth media (GM) and counted on day 4 of proliferation (Figure 1). All treated cells showed a spindle-like shape, which is common in proliferating satellite cell morphology [21], and a higher cell density was observed in p38i-treated cultures than in control cultures (Figure 1A). Cells treated with p38i had higher numbers of proliferating cells compared to those in control cells throughout all passages (p<0.05). In particular, 10 μM p38i-treated cultures had the highest number of cells (Figure 1B). This suggests that treatment with p38i may enhance the proliferation of primary chicken muscle stem cell cultures.

### Effect of p38i on the maintenance of myogenic marker gene expression

**Figure 2:**
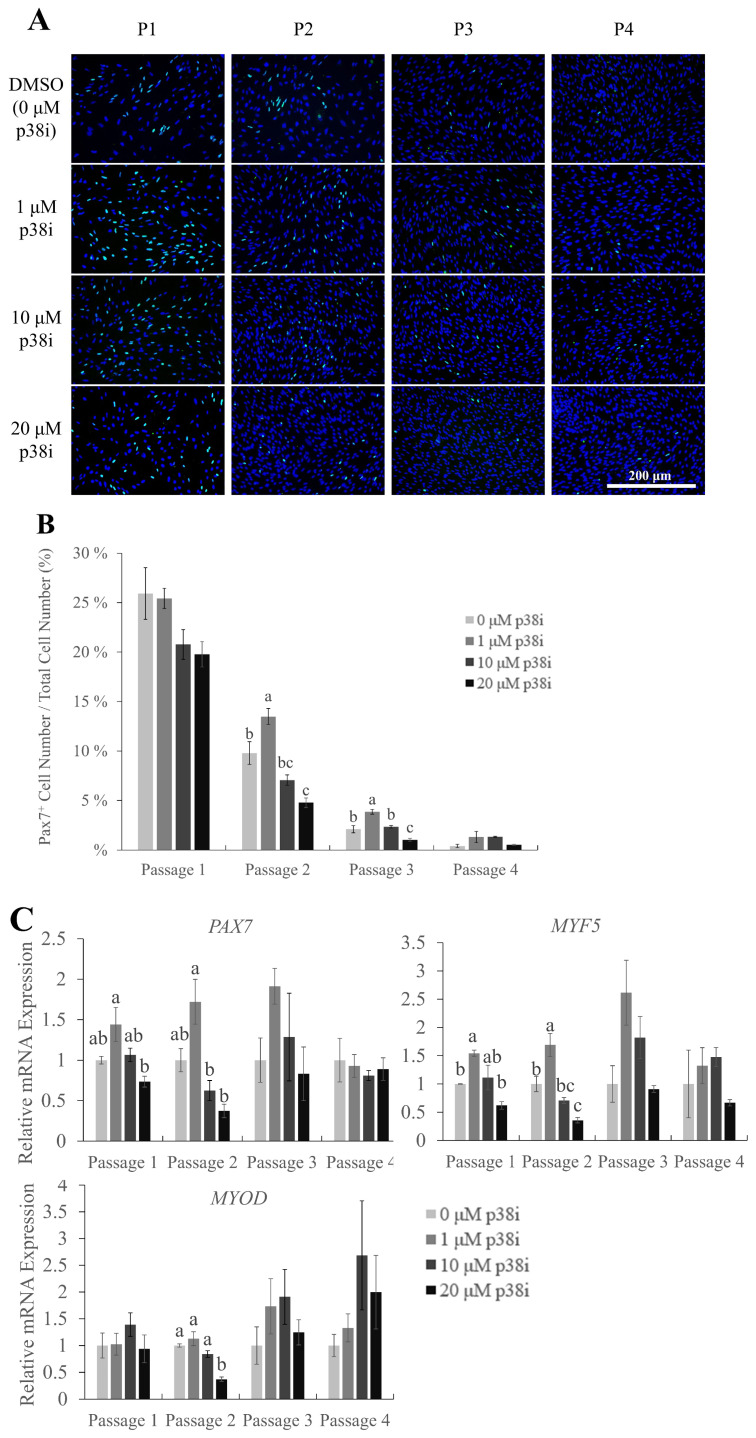
Effect of p38i treatment on myogenic marker gene expression. (A) Immunofluorescence staining of Pax7 (green) in control and p38i-treated cell cultures. Nuclei were stained with Hoechst (blue). (B) Percentage of Pax7^+^ cells in control and p38i-treated cell cultures. Cells were counted and stained on day 4 of proliferation. (C) Relative mRNA expression of proliferating myogenic markers *PAX7*, *MYF5*, and *MYOD* in muscle stem cells on day 4 of each passage. All mRNA expression levels were normalized to *GAPDH* expression levels. All passages of each replication originated from the same primary cell line. p38i, p38 inhibitor; *PAX7*, paired box 7; *MYF5*, myogenic factor 5; *MYOD*, myoblast determination protein 1; *GAPDH*, glyceraldehyde-3-phosphate dehydrogenase. ^a–c^ Different letters within the same passage number indicate significant differences (p<0.05). Scale bar = 200 μm. Effect of p38i treatment on myogenic marker gene expression. (A) Immunofluorescence staining of Pax7 (green) in control and p38i-treated cell cultures. Nuclei were stained with Hoechst (blue). (B) Percentage of Pax7^+^ cells in control and p38i-treated cell cultures. Cells were counted and stained on day 4 of proliferation. (C) Relative mRNA expression of proliferating myogenic markers *PAX7*, *MYF5*, and *MYOD* in muscle stem cells on day 4 of each passage. All mRNA expression levels were normalized to *GAPDH* expression levels. All passages of each replication originated from the same primary cell line. p38i, p38 inhibitor; *PAX7*, paired box 7; *MYF5*, myogenic factor 5; *MYOD*, myoblast determination protein 1; *GAPDH*, glyceraldehyde-3-phosphate dehydrogenase. ^a–c^ Different letters within the same passage number indicate significant differences (p<0.05). Scale bar = 200 μm.

Pax7 is a key myogenic regulatory factor (MRF) that is characteristically expressed in muscle stem cells to maintain their self-renewal abilities. The expression of Pax7 gradually decreases as myogenic differentiation occurs [8,22,23]. Immunofluorescent staining images of Pax7 in chicken muscle stem cell cultures showed a rapid decrease in the percentage of Pax7-expressing (Pax7^+^) cells as the cells were subcultured (Figure 2A). The percentage of Pax7-positive cells to the total number of cultured cells was the highest in 1 μM p38i-treated cultures at passages 2 and 3, followed by 10 μM p38i-treated and control cultures (Figure 2B, p<0.05). Relative mRNA expression levels of *PAX7* and *MYF5* were also significantly higher in the 1 μM p38i-treated group than in the other groups up to passage 2. However, the expression of *MYOD* was not significantly affected by p38i treatment, except for a decrease in its expression in 20 μM-treated cells at passage 2.

These results indicate that moderate treatment with p38i may help maintain the stemness of chicken muscle stem cells. However, its effect on long-term culture for over three passages needs to be studied further.

### Effect of p38i on the myogenic differentiation of chicken muscle stem cells

**Figure 3:**
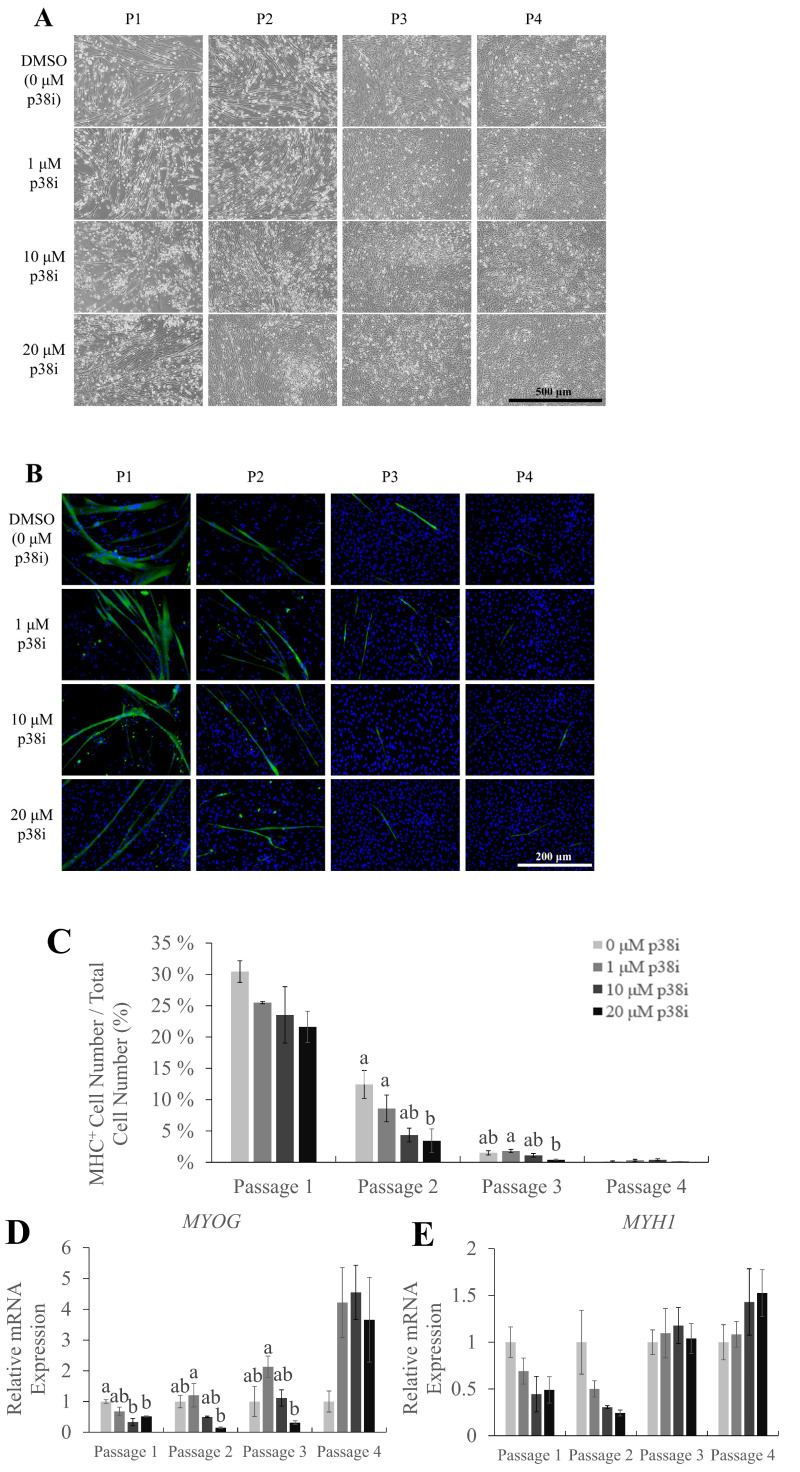
Myogenic potential of chicken muscle stem cells after treatment with p38i. (A) Morphology of cells on day 3 of differentiation. (B) Immunofluorescence staining of myosin heavy chain (MHC, green). Nuclei were stained with Hoechst (blue). (C) Percentage of MHC^+^ cells in control and p38i-treated cell cultures. (D), (E) Relative mRNA expression of myogenic differentiation markers (D) *MYOG* and (E) *MYH1*. All mRNA expression levels were normalized to *GAPDH* expression levels. Cells were allowed to proliferate up to day 4 and then allowed to differentiate in differentiation medium for 3 days. All passages of each replication originated from the same primary cell line. *MYOD*, myoblast determination protein 1; *MYH1*, myosin heavy chain 1; *GAPDH*, glyceraldehyde-3-phosphate dehydrogenase. ^a,b^ Different letters within the same passage number indicate significant differences (p<0.05). Scale bar = 500 μm (A), 200 μm (B). Myogenic potential of chicken muscle stem cells after treatment with p38i. (A) Morphology of cells on day 3 of differentiation. (B) Immunofluorescence staining of myosin heavy chain (MHC, green). Nuclei were stained with Hoechst (blue). (C) Percentage of MHC^+^ cells in control and p38i-treated cell cultures. (D), (E) Relative mRNA expression of myogenic differentiation markers (D) *MYOG* and (E) *MYH1*. All mRNA expression levels were normalized to *GAPDH* expression levels. Cells were allowed to proliferate up to day 4 and then allowed to differentiate in differentiation medium for 3 days. All passages of each replication originated from the same primary cell line. *MYOD*, myoblast determination protein 1; *MYH1*, myosin heavy chain 1; *GAPDH*, glyceraldehyde-3-phosphate dehydrogenase. ^a,b^ Different letters within the same passage number indicate significant differences (p<0.05). Scale bar = 500 μm (A), 200 μm (B).

To investigate the effects of p38i treatment on muscle differentiation and proliferation, myogenic differentiation marker expression was analyzed. The myosin heavy chain is a part of myosin, a family of proteins responsible for muscle contraction. In myogenesis, MHC is expressed when myoblasts differentiate and form myofibers [24]; thus, it is commonly used as a marker for late myogenesis. Immunofluorescence staining of MHC on day 3 of differentiation showed the presence of multinucleated myotubes, which decreased in number and size as cells were subcultured, throughout all treatments (Figure 3A–B). Treatment with p38i did not increase the percentage of MHC-expressing cells, except in passage 3, where the 1 μM group had a significantly higher percentage of MHC-expressing cells compared to the 20 μM group. (Figure 3C; p<0.05). Moreover, the expression of myosin heavy chain 1 gene (*MYH1*) was not significantly different between the treatments (Figure 3E). However, *MYOG* was expressed at significantly higher levels in 1 μM p38i-treated cell cultures (Figure 3D).

### Accumulation of lipid in p38i-treated cell cultures

**Figure 4:**
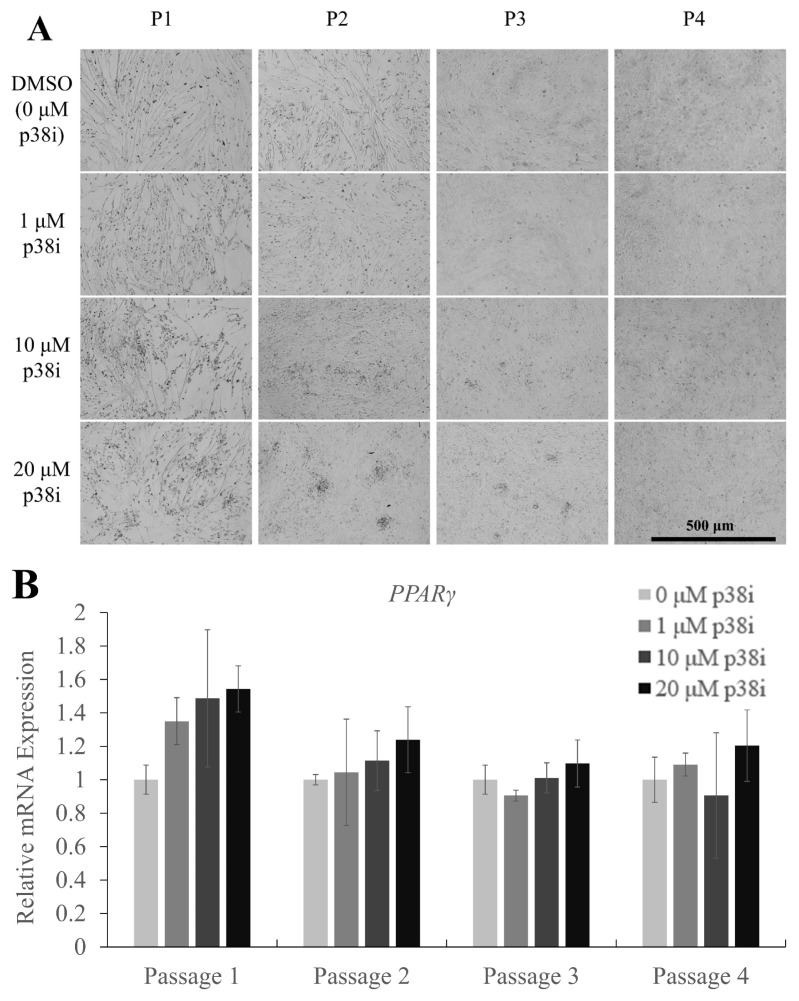
Adipogenic marker expression and lipid accumulation in p38i-treated chicken muscle stem cells. (A) Oil red O staining of lipid droplets in control and p38i-treated cell cultures. (B) Relative mRNA expression of the adipogenic differentiation marker *PPARγ*. All mRNA expression levels were normalized to those of *GAPDH* expression levels. Cells were allowed to proliferate up to day 4 and then allowed to differentiate in differentiation medium for 3 days. All passages of each replication originated from the same primary cell line. Scale bar = 500 μm. p38i, p38 inhibitor; *PPARγ*, peroxisome proliferator activated receptor gamma; *GAPDH*, glyceraldehyde-3-phosphate dehydrogenase.

After the media replacement with differentiation medium (DM), lipid droplet-like structures were observed in some cell cultures. To confirm whether adipogenesis was induced, cells on day 3 of differentiation were stained with oil red O to visualize lipid droplets (Figure 4A). Images showed a higher density of stained droplets in p38i-treated cultures, especially in the 10 and 20 μM treatment groups. Relative mRNA expression of the adipogenic differentiation marker gene peroxisome proliferator activated receptor gamma (*PPARγ*) showed a tendency to increase in a p38i concentration-dependent manner, but statistical analysis revealed no significant upregulation in any of the treatments (Figure 4B).

## DISCUSSION

The p38-MAPK pathway regulates myogenesis by signaling myogenic differentiation and downregulating Pax7 expression. Thus, inhibition of this pathway leads to the maintenance of Pax7 expression and stemness of muscle stem cells, as shown in previous studies [10,11]. Prolonged stemness of muscle stem cells and the subsequent increase in self-renewal and proliferation are important for muscle regeneration and efficient cultured meat production. The results of this study were similar to those of previous studies, as p38i treatment led to increased Pax7 and Myf5 expression, which indicates the increase of cells that maintain the cell cycle and proliferate to produce cells that may differentiate into cells of the myogenic lineage [22].

The p38 inhibitor (SB203580) used in this study is known to inhibit p38α and p38β of the p38-MAPK family. Previous studies on the p38-MAPK pathway and myogenesis indicate that myoblasts lacking p38α undergo continuous proliferation, and thus are unable to differentiate and form multinucleated myotubes even in differentiation-promoting conditions [25]. Consistent with these results, while wild-type myoblasts showed reduced activation of the JNK signaling pathway, p38α-deficient myoblasts maintained JNK activity, which in turn increased myoblast proliferation. Moreover, p38α-deficient myoblasts also had reduced myofiber growth in the same study, but not through the JNK pathway. Thus, we believe that the p38i used in this study affected the myogenic differentiation of chicken muscle stem cells through a similar mechanism.

However, the effect of p38i on long-term culture needs to be investigated. In this study, the effect of p38i treatment seemed to last only for a few passages, and the differences between the treatment and control groups were not dramatic although the significance was verified. This may be due to differences in culture conditions. The culture medium used in this study contained minimal supplements of 10% FBS, Glutamax, and no growth factors. In previous studies, supplementation with fibroblast growth factor or fibroblast growth factor 2 [21,26,27], epidermal growth factor, and dexamethasone [10] have been used for the stimulation of proliferation. Mixtures of growth factors such as chicken embryo extract have been shown to enhance myoblast differentiation [28]. Thus, in future studies, the use of such myoblast culture supplements may maintain myoblasts for a longer period, thus allowing one to study the effects of the treatments effectively.

The differentiation ratio, shown by the expression of the myogenic differentiation marker MHC, was not significantly different between the two groups. Compared to control cells, in p38i-treated cultures, the mRNA expression of MHC was decreased, unlike the results of previous studies on p38 inhibition in muscle stem cells [10,11]. *MYOG*, an MRF that regulates myogenic cell fusion, is known to initiate differentiation at the early stages of myogenic differentiation [24], while MHC isotypes indicate late myogenesis. Therefore, one explanation of these results may be that the myoblasts on day 3 of differentiation were not mature enough for the expression of late differentiation marker MHC. The expression of *MYOG*, however, indicates the presence of cells that have committed to the myogenic lineage, thus proving that 1 μM p38i treatment could maintain the myogenic differentiation potential of chicken muscle stem cells nonetheless. There is also a possibility that the p38-MAPK signaling pathway was not recovered even after the removal of p38i. Therefore, the myogenic differentiation process was continuously suppressed, blocking the expression of MHC. Further investigation on the activation of the p38-MAPK pathway throughout the proliferation and differentiation of p38i-treated muscle stem cells may be needed. In turn, this suppression of myogenic differentiation may have induced lipid accumulation.

Previous studies have shown that myogenesis, especially the expression of MyoD, inhibits adipocyte differentiation [29]. However, in this study, the accumulation of lipid droplets was observed in differentiated muscle stem cell cultures, which was in disagreement with the results of previous studies. Moreover, despite no significant differences in *PPARγ* expression levels, its expression tended to increase in p38i concentration-dependent manner. From these results, lipid accumulation seems to be induced by p38i treatment, but further analysis is needed to confirm adipogenic differentiation. Supporting this outcome, there have been reports on the transdifferentiation of myogenic cell cultures into adipocytes or adipose-like cells, which demonstrate lipid accumulation in myogenic lineage cells [19,30,31]. Therefore, the specific proliferation or differentiation conditions used in this experiment may have triggered spontaneous transdifferentiation of chicken muscle stem cell cultures, as in the above studies.

Another possibility is that incomplete purification of muscle stem cells led to the proliferation and adipogenic differentiation of non-myogenic cells, such as mesenchymal stem cells. Methods such as fluorescence-activated cell sorting, magnetic-activated cell sorting, Percoll density centrifugation, and pre-plating are generally used for the isolation of muscle stem cells; however, none of these methods can be used for 100% isolation. Mesenchymal stem cells are multipotent stem cells that can act as precursors of both myoblasts and adipocytes in muscle cells. Therefore, the infiltration of mesenchymal stem cells may have been the source of adipogenic cultures [32]. However, mesenchymal stem cells co-cultured with myoblasts undergo myogenic differentiation, whereas adipogenic differentiation requires specific conditions that differ from those of myogenic differentiation [33]. Thus, it is unclear how lipid accumulation was induced in muscle stem cell cultures. Further studies on the relationship between the p38-MAPK pathway and adipogenesis in chicken muscle stem cells are needed.

## CONCLUSION

In this study, treatment with p38i increased the proliferative ability of chicken muscle stem cells. The increase in cell number and expression of myogenic marker genes was significantly higher in p38i-treated cultures than in control cultures. While the myogenic differentiation abilities of chicken muscle stem cells did not improve after inhibition of the p38 pathway compared to those in the control group, p38i-treated muscle stem cells exhibited a higher number of lipid droplets than control cells. This investigation of adipogenic differentiation in muscle stem cell cultures supports new possibilities in the cultured meat industry. This may lead to the co-culture of myotubes and adipose tissue, allowing for the efficient production of cultured meat with properties similar to those of conventional meat, which is composed of muscle and other components, including fat. However, as stated above, further studies are required to determine whether adipocyte differentiation can be induced in chicken muscle stem cells.

## Figures and Tables

**Table 1:** Primers used for quantitative reverse-transcription polymerase chain reaction

Gene	Primer sequence (5′ → 3′)		Product size (bp)	Accession No.
*PAX7*	Forward	CTCCTGCCAACCACATGAAT	108	NM_205065.1
	Reverse	TGTTCAAGGCTATGGTGAGGTT		
*MYF5*	Forward	ACCAGAGACTCCCCAAAGTG	106	NM_001030363.1
	Reverse	CCCGGCAGGTGATAGTAGTTC		
*MYOD*	Forward	CGCAGGAGAAACAGCTACGA	146	L34006.1
	Reverse	GTGACTGGGGACAGAGAAGG		
*MYOG*	Forward	ATGGGGAAAACTTCCTGGGC	109	NM_204184.1
	Reverse	TTCTCCTCCAAAGCCCCTCT		
*MYH1*	Forward	TCCGCAAGATCCAACACGAA	150	NM_001013397.2
	Reverse	ATGCCACTTTGTTGTCACGA		
*PPARγ*	Forward	CTCCAGGATTGCCAAAGTGC	138	NM_001001460.1
	Reverse	GTCCCCACACACACGACATT		
*GAPDH*	Forward	CGTCCTCTCTGGCAAAGTCC	132	NM_204305.1
	Reverse	TTCCCGTTCTCAGCCTTGAC		

*PAX7*, paired box 7; *MYF5*, myogenic factor 5; *MYOD*, myoblast determination protein 1; *MYOG*, myogenin; *MYH1*, myosin heavy chain 1; *PPARγ*, peroxisome proliferator activated receptor gamma; *GAPDH*, glyceraldehyde-3-phosphate dehydrogenase.
